# Non-Cardiac Cause of Death in Selected Group Children with Cardiac Pathology: A Retrospective Single Institute Study

**DOI:** 10.3390/children9030335

**Published:** 2022-03-02

**Authors:** Stefana Maria Moisa, Ingrith Crenguta Miron, Elena Tarca, Laura Trandafir, Vasile Valeriu Lupu, Ancuta Lupu, Tania Elena Rusu

**Affiliations:** 1 Pediatrics Department, “Grigore T. Popa” University of Medicine and Pharmacy, 700115 Iasi, Romania; stephaniemed@yahoo.com (S.M.M.); ingridmiron@hotmail.com (I.C.M.); elatarca@gmail.com (E.T.); trandafirlaura@yahoo.com (L.T.); 2 Independent Researcher, 85200 Kalymnos, Greece; taniamavrou@yahoo.com

**Keywords:** dilatative cardiomyopathy, hypertrophic cardiomyopathy, cardiomegaly, children, mechanism of death

## Abstract

Background: Pediatricians and pediatric surgeons often face children with cardiomegaly and dilatative or hypertrophic cardiomyopathies presenting with or without symptoms. Some of these patients have already been diagnosed and received medication, and some present with completely unrelated pathologies. Methods: We performed a 4-year retrospective study on the causes and mechanisms of death of children with cardiac pathology who died outside the cardiology clinic of our hospital by studying the hospital charts and necropsy reports. All children who were in this situation in our hospital were included. Results: Most children in our study group were infants (81.82%), most were boys (81.82%), and in most cases, the cause or mechanism of death was unrelated to their heart condition, whether it had already been diagnosed or not (one case probably died as a result of a malignant ventricular arrhythmia). Additionally, 27.27% of children died as a consequence of bronchopneumonia, the same percentage died as a consequence of an acquired non-pulmonary disease or after surgery, and 18.18% died as a consequence of congenital malformations. Conclusions: Cardiac disease needs to be thoroughly investigated using multiple tools for all children presenting with heart failure symptoms, those with heart murmurs, and children scheduled for surgery of any type. The intensive care specialist and surgeon need to be aware of any heart pathology before non-cardiac surgical interventions.

## 1. Introduction

Of all children born worldwide, 0.8–1.2% suffer from congenital cyanotic or non-cyanotic heart malformations [1]. Furthermore, acquired heart diseases, such as dilated cardiomyopathy secondary to myocarditis; iron overload; arrhythmia; electrolyte imbalances; autoimmune or endocrinological diseases; nutritional deficiencies, drug exposure, or hypertrophic cardiomyopathy secondary to storage diseases; fatty acid oxidation abnormalities; primary hyperinsulinism or acromegaly; and valvulopathies, are also possible, making heart disease an important source of mortality and morbidity in all pediatric age categories. When other severe conditions, either medical or surgical, overlap, the prognosis is grim, but the cause or mechanism of death is not always related to the heart disease itself.

Our study aimed to identify the noncardiac causes of death of children suffering from cardiomyopathies or cardiomegaly in order to improve the management of these cases. This topic is seldom addressed in the literature; a search for the keywords “noncardiac causes of death dilated cardiomyopathy cardiomegaly” performed on PubMed yielded no significant results, and articles on dilated cardiomyopathy or cardiomegaly focus on cardiac disease evolution and prognosis [2,3].

## 2. Materials and Methods

We performed a retrospective study on all children diagnosed with any type of cardiomyopathy or cardiomegaly due to heart malformations who died outside the cardiology clinic (in other clinics) of Saint Mary Emergency Children’s Hospital in Iasi, Romania, over a 4-year period (2018–2021). This study has no selection bias, as we included all patients suffering from various conditions who died outside the cardiology clinic in the given timeframe. We examined the files and necropsy reports of these children. Clinical files included reasons for admission; information about the clinical examination of the patient upon admission and daily evolution; hematological and biochemical parameters; imaging results (X-ray, echocardiogram, etc.); and medical, intensive care, and surgical treatment.

The necropsy files included data about the general inspection of the corpse, macroscopic analysis of the internal organs (including assessments of the heart size and type of heart malformation), and, in most cases, microscopic organ analysis (including that of the myocardium).

Informed consent was obtained from all parents or caregivers at the time of admission, and the Saint Mary Children’s Emergency Hospital Ethics Committee’s approval was obtained for publishing this study (19077/2 July 2021).

We identified 11 children suffering from heart conditions during a 4-year period who died in non-cardiology clinics of our hospital. Some children died on the same day they were admitted to our hospital, some had been suffering from cardiomyopathies or heart malformations leading to cardiomegaly for some time, and some were first diagnosed during the admission in which they passed away; for others, the cardiomegaly or cardiomyopathy diagnose has been established after death.

Of the 11 cases analyzed, 2 were female and 9 were male (see Table 1).

## 3. Results

Patient number 1 was transferred from a local maternity ward with the suspicion of duodenal stenosis. The child was prematurely born at home at the gestational age of 32 weeks and had a birth weight of 1500 g. He was admitted into a newborn intensive care facility 2 h after birth. Upon admission, signs of respiratory failure were present, and there was a III/VI systolic murmur audible over the precordial area. Echocardiography initially showed the presence of a large interatrial septal defect, a ventricular septal defect, and a patent ductus arteriosus. The presence of a moderate form of coarctation of the aorta was later diagnosed. Two months after birth, the cardiologist diagnosed global cardiac hypertrophy (concentric left ventricle hypertrophy, right atrial and right ventricular hypertrophy), and one month later, pulmonary hypertension was also present. The corpus callosum agenesis was diagnosed on the second day of life. A cardiac surgery center was contacted, but surgery was not performed because the case did not represent a cardiac surgery emergency.

The evolution of the case was unfavorable. The patient received oxygen beginning on the first day of life and was intubated at the age of 2 months. Diuretic therapy for heart failure was started on day 34. A few days later, several episodes of vomiting appeared; as they became more and more frequent, a barium meal raised the suspicion of duodenal stenosis, and the patient was admitted to the general surgery ward of our hospital. He needed to be resuscitated as soon as he arrived, and the clinical status did not improve enough during the hospitalization to allow surgery to be performed.

Patient number 2 was born in the 37th week of gestation and had a birth weight of 2550 g and an APGAR score of 4 at 1 min. The mother had previously given birth to another boy suffering from spinal amyotrophy who died 3 months after birth. The child needed to be intubated from birth because of inefficient spontaneous respiration. The evolution was unfavorable, with bronchoplegia, generalized hypotonia, ascension of the right hemidiaphragms, absence of osteotendinous, and archaic reflexes. Echocardiography showed a large patent foramen ovale; septal hypertrophic cardiomyopathy; and dilatation of the right atrium, right ventricle, and pulmonary artery. The child did not undergo surgery, but a center specializing in treating spinal amyotrophy was contacted for transfer.

Patient number 3 was known to suffer from obstructive hypertrophic cardiomyopathy, moderate aortic stenosis, ventricular septal defect, Wolff–Parkinson–White preexcitation syndrome, partial corpus callosum agenesis bilateral hip dysplasia, and posterior palatoschisis. He was admitted to the general surgery ward of our hospital for surgical treatment of umbilical and inguinal hernias. The procedure was performed with no incidents, but the patient died 2 h after surgery.

Patient number 4 was suffering from tetralogy of Fallot with severe pulmonary stenosis, severe tricuspid regurgitation, patent ductus arteriosus, and class III NYHA heart failure and was receiving cardiological medication. At the time of the cardiorespiratory arrest, he was admitted to an external ward of our hospital. Resuscitation was initiated and later continued by the ambulance personnel, but the outcome was unfavorable. The necropsy established the diagnosis of transposition of the great arteries, a ventricular septal defect, patent ductus arteriosus, and minimal aortic stenosis.

Patient number 5 had two cases of malignant hematological diseases in his family. At the age of 6 weeks, the parents presented to the hospital because the child was agitated and refused feedings. The hemoleucogram showed pancytopenia, and the child was transferred to our hospital, where the diagnosis of hemophagocytic lymphohistiocytosis was established on the basis of the presence of hepatosplenomegaly, severely increased ferritin, hepatic dysfunction, severe hypofibrinogenemia, hypertriglyceridemia, hypoproteinemia, hyponatremia, jaundice, edema, skin rash, and exclusion of other hematological malignancies by marrow immunophenotyping. Echocardiography showed a small patent foramen ovale, and the necropsy also diagnosed hypertrophic cardiomyopathy.

Patient number 6 was admitted by transfer from a smaller hospital, where he had been hospitalized for one day for signs of respiratory distress, ineffective cough, agitation, and vomiting. This was a child also suffering from cow milk protein intolerance and gastroesophageal reflux. Upon admission to our hospital, a second-degree systolic murmur was noticed, and the cardiology consult established the diagnosis of dilated cardiomyopathy and class IV Ross heart failure. In this case, the dilated cardiomyopathy was probably secondary to a viral myocarditis episode, as the mother noticed progressive effort dyspnea following a diarrhea episode. The chest X-ray was suggestive of bronchopneumonia. The child died despite antibiotic, supportive, and positive inotropic treatment.

Patient number 7 was suffering from chronic infantile encephalopathy, spastic tetraparesis, hydrocephaly, and atrial septal defect. He developed signs and symptoms of an inferior respiratory infection whose evolution was unfavorable. The anatomopathological exam also discovered the presence of dilated cardiomyopathy.

Patient number 8 was diagnosed 10 months earlier with third-degree posterior fossa ependymoma involving the cerebral trunk, cerebellum, and fourth ventricle and was a ventriculoperitoneal and tracheostomy carrier with no neurosurgical indication. During hospitalization, he became febrile; purulent secretion was drained from the tracheostomy; the oxygen saturation dropped despite oxygen, antibiotic, and supportive treatment; fixed miosis appeared; and irreversible cardiorespiratory arrest occurred. The necropsy described the presence of cardiomegaly.

Patient number 9 was suffering from Prader–Willy syndrome, epilepsy, severe obesity, severe neuromotor retardation, had a seeing and hearing impairment, and had been hospitalized for 6 days before admission to a smaller hospital, where he received treatment for bronchopneumonia. He was oxygen-dependent during the entire hospitalization at our hospital, despite intensive antibiotic, pathogenic, and supportive treatment. The necropsy discovered cardiomegaly, aortic and pulmonary artery enlargement, and a vascular abnormality—common origin of the brachiocephalic trunk and primitive left carotid artery.

Patient number 10 was born in the 36th week of gestation and was diagnosed postpartum with giant omphalocele accompanied by intestinal malrotation and occlusion. She was admitted to our hospital for surgical management. The first part of the surgery proceeded smoothly; the liver, spleen, and part of the intestine were integrated into the peritoneal cavity, and 10 days later, the rest of the intestine was integrated, and the abdominal wall was sutured. Soon after surgery, the patient developed edema, thrombocytopenia, and cardiocirculatory arrest. Necropsy findings included cardiomegaly.

The last patient was born during the 30th week of gestation and diagnosed after birth with spina bifida. She received neurosurgical treatment for this condition, but the postoperative evolution was unfavorable. Because of the urgency of the neurosurgical intervention, this child was not examined by a cardiologist before intervention. The necropsy showed that she was suffering from truncus arteriosus and a membranous ventricular septal defect causing predominant right-sided heart enlargement.

The average hospitalization duration was 12.09 days and ranged from 1 day to 31 days. In two cases, the cardiac diagnosis was established via echocardiography and did not coincide with necropsy findings.

All but two children were newborns or infants at the time of death. Two out of eleven children were diagnosed with a heart condition before their last hospitalization, and they were receiving cardiac medication. In three cases, the heart condition was first diagnosed during necropsy. Three children underwent surgery for non-cardiac conditions (umbilical and inguinal hernia, giant omphalocele, intestinal malrotation, and occlusion and spina bifida with myelomeningocele). The deaths of these patients occurred in the postoperative period, and the mechanism of death was probably related to periprocedural events or anesthetic procedures.

Four out of eleven children died as a consequence of their main disease: corpus callosum agenesia, hemophagocytic lymphohistiocytosis, spinal amyotrophy, or brain tumor. Additionally, 27.27% of children died as a consequence of bronchopneumonia, the same percentage died as a consequence of an acquired non-pulmonary disease or after surgery, and 18.18% died as a consequence of congenital malformations. One child died as a result of a probable malignant ventricular arrhythmia (sudden death).

The echocardiographic examination was performed using a General Electric machine. The images below were obtained from a dilated cardiomyopathy patient.

**Figure 1:**
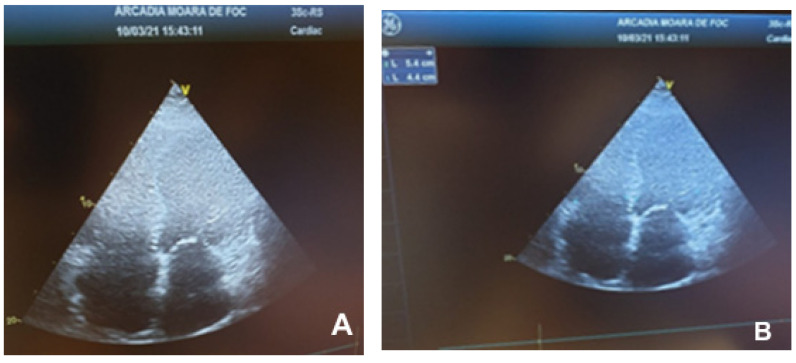
Dilated cardiomyopathy echocardiogram. (**A**,**B**)—Apical 4 chamber view with global cardiomegaly and abnormal sphericity index.

**Figure 2:**
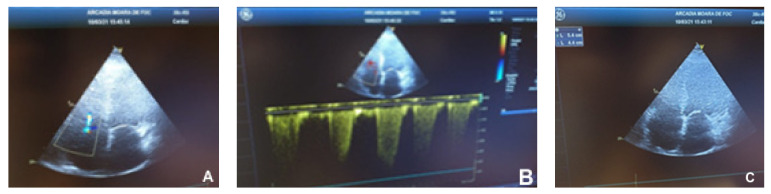
Dilated cardiomyopathy echocardiogram. (**A**)—Mild tricuspid regurgitation allowing the measurement of (**B**)—right ventricular–right atrial gradient and the estimation of systolic pulmonary artery pressure. (**C**)—diminished tricuspid annular plane systolic excursion.

**Figure 3:**
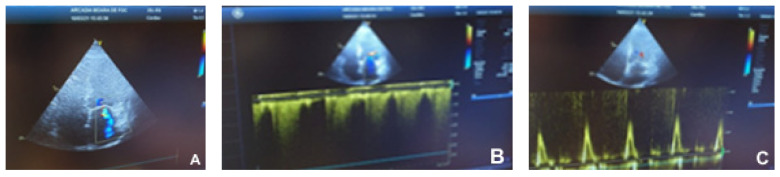
Dilated cardiomyopathy echocardiogram. (**A**)—Severe mitral regurgitation (apical four-chamber); (**B**)—tricuspid envelope continuous Doppler; (**C**)—trans-mitral pattern that is suggestive of atrial fibrillation.

Marked ventricular remodeling caused the sphericity index to become abnormal (Figure 1). Low cardiac output was suggested by decreased amplitude and duration of mitral and aortic valve opening. Mitral (Figure 2) and tricuspid (Figure 3) regurgitations ranging from mild to severe may have existed.

The pediatric cardiologist should remember to check for the presence of atrial, appendage or ventricular thrombi, or spontaneous contrast. The likelihood of discovering such masses is proportional to the severity of the systolic dysfunction. In addition, the existence of pulmonary hypertension should be checked upon the first presentation and later during the evolution of the disease.

**Figure 4:**
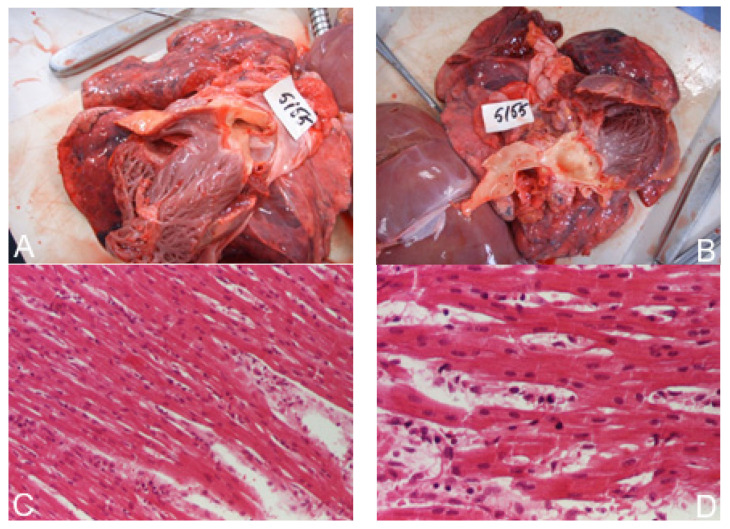
Dilated cardiomyopathy. (**A**,**B**)—macroscopic aspect, (**C**)—myocarditis: hematoxylin–eosin × 100, (**D**)—myocarditis: hematoxylin–eosin × 200.

Necropsy included macroscopic and microscopic examination of the heart (Figure 4). Myocarditis was found in several cases was associated with bronchopneumonia.

## 4. Discussion

A total of 74,053 children were treated in the hospital during the 4 years we studied (2018–2021). There were 358 deaths reported during this period in our hospital (0.48%) (Table 2).

The patients in our study group suffered either from congenital heart malformations leading to cardiomegaly or cardiac hypertrophy or from primary cardiomyopathies—dilated or hypertrophic. However, their deaths were not caused by their heart conditions alone in all but one case, who probably developed a malignant ventricular arrhythmia, which manifested as sudden death, in a patient with transposition of the great arteries and a patent ductus arteriosus.

Severe inferior respiratory disease, such as bronchopneumonia, seems to have a worse prognosis for infants suffering from cardiac malformations with pulmonary over-circulation [4], despite maximal intensive care treatment directed at the heart failure and respiratory disease itself. The patients diagnosed with bronchopneumonia in our group, however, did not have left–right shunt malformations. Two developed cardiac chamber dilatations as a consequence of dilatative cardiomyopathy, and one suffered from hypertrophic cardiomyopathy.

None of the children were cyanotic during hospitalization. Children directly admitted to a surgical ward did not always have a cardiology consult before their operation, and neither did one child who was admitted to a general pediatrics clinic. All children admitted to the intensive care unit, however, did undergo a cardiology consultation. A systolic murmur was only described upon admission for three children, but not for the one suffering from one of the most severe malformations—truncus arteriosus.

Our study group was mainly composed of infants and male children. This is consistent with other group findings, as both hypertrophic [5] and dilated [6] cardiomyopathies were found to have a higher prevalence in males.

## 5. Current Knowledge Status

### 5.1. Definitions

Dilated cardiomyopathy is defined by the presence of left ventricular or biventricular dilatation and systolic dysfunction (accompanied by diastolic dysfunction or not) in the absence of other causes of systolic impairment, such as sepsis, abnormal loading (valvular diseases or hypertension), and coronary artery disease. In children, ventricular dilatation should be diagnosed in the presence of an end-diastolic volume or diameter larger than +2 standard deviations corrected for body surface area, age, and gender [7,8]. A Z score above 2 applied to left ventricular end-diastolic or end-systolic volumes or diameters is relevant for the diagnosis [9].

Hypertrophic cardiomyopathy is defined as an increase in the heart wall thickness above the normal values for age, height, and weight that cannot be explained only by abnormal ventricular load. In children, this condition has an important genetic predisposition [10] involving the actin, myosin, tropomyosin, and titin genes [11].

### 5.2. Incidence

Dilated cardiomyopathy incidence in the United States of America seems to be 1.13 cases in 100,000 children [12], similar to what one study [13] found in Australia—1.24 in 100.000—and the findings of another study [14] in Finland—0.34 cases per 100,000 children per year. One study conducted in New England and the Central Southwest reported an annual incidence of 1.13 per 100,000 infants and children [12]. Another study conducted in Egypt and Kuwait reported an overall rate of dilated cardiomyopathy in childhood of 0.07 cases/100,000/year [15].

The peak incidence of the disease is in the first two years of life [16], and this pathology represents the most frequent indication for heart transplantation in children.

One study found that 42% of all childhood cardiomyopathies are of the hypertrophic type, and the incidence of the disease is 0.47 in 100.00 children [12].

### 5.3. Etiology

The causes of dilated cardiomyopathy in childhood are mainly genetic, involving sarcomere mutations or mutations of nuclear proteins, ion channel proteins, or cytoskeletal proteins [17]. Other causes include infectious causes (viral, bacterial, fungal, or parasitic), iron overload [18], electrolyte imbalances, endocrinological causes, nutritional deficiencies (carnitine, selenium, or thiamine), autoimmune diseases (polymyositis), drug exposure (antineoplastics or antipsychotics), and tachycardia-induced cardiomyopathy [4].

Anthracyclines (daunorubicin or doxorubicin) are well-known to induce dose-dependent cardiac toxicity, manifesting as congestive heart failure during or immediately after treatment is completed. Children receiving more than 300 g/m^2^ of anthracyclines were found to have increased left ventricular afterload, sometimes accompanied by contractility impairment [19]. Cardiotoxicity was more severe in females, in children receiving high doses of daunorubicin or doxorubicin, and in those also receiving cyclophosphamide or spinal or mediastinal irradiation for acute leukemia or lymphoma [20].

Various infectious agents are responsible for myocarditis, which may progress to dilated cardiomyopathy. During the last two decades, coxsackieviruses were the most frequently implicated viral agents [21], but today, the attention has shifted to Parvovirus B19, human herpesvirus type 6, and novel coronavirus SARS-CoV2 [22,23]. Other known causes of viral myocarditis include Epstein Barr [24], cytomegalovirus [25], adenovirus [26], hepatitis C virus [27], parainfluenza, H1N1 influenza A virus [28], and human immunodeficiency virus [29]. Bacterial agents causing myocarditis and, in some cases, subsequently dilated cardiomyopathy include Clostridium [30], Diphtheria [31], and parasites, such as Trypanosoma cruzi [32], Toxocara [33], Toxoplasma [34], and Cysticercus [35], have also been incriminated for the disease. Fungal myocarditis caused by Histoplasma [36], Actinomyces [37], or coccidioidomycoses [38] can also progress to dilated cardiomyopathy.

Not only infectious causes are incriminated in the development of dilated cardiomyopathy in children. Most children affected by muscular dystrophy eventually develop low cardiac output symptoms as a sign of heart dilatation and systolic impairment, ultimately causing death. Duchene and Becker muscular dystrophies, both caused by mutations in the dystrophin gene, are characterized by abnormal cardiomyocyte function in terms of ion channel stretch response. Stretch causes increased cardiomyocyte calcium influx, which activates proteases that degrade troponin I [39]. Chronic calcium overload causes cardiomyocyte apoptosis [40]. Although initially, children with Friedreich’s ataxia have echocardiography findings of hypertrophic cardiomyopathy, toward the end of their lives, they also present with features of dilated cardiomyopathy. In this stage, they develop life-threatening arrhythmia and abrupt heart function decline [41]. Types I, II, and VI mucopolysaccharidoses are sometimes associated with dilated cardiomyopathy, although valve disease and not chamber dilatation is a more specific association [42]. More typical dilated cardiomyopathy echocardiography findings are found in glycogen storage disease children (Danon disease, phosphorylase kinase deficiency, Brancher enzyme deficiency, and Glycogenin-1 deficiency) [43].

Dilated cardiomyopathy can be caused by several endocrine diseases, such as pheochromocytoma [44], hypothyroidism [45], hyperthyroidism [46], and hypoparathyroidism [47]. More specific etiologies for dilated cardiomyopathy in newborns, infants, and children refer to lysosomal diseases, glycogen storage disorders, mitochondrial disorders, fatty acid oxidation disorders, and organic acidurias. Fatty acid oxidation disorders should be suspected in children with cardiomyopathy who develop recurrent episodes of nonketotic hypoglycemia. A hypotonic child with dilated cardiomyopathy should be suspected to have Pompe disease, while all storage diseases should be investigated in a child with dilated cardiomyopathy and hepatomegaly [48].

Ischemic dilated cardiomyopathy in children is rare and is due to ALCAPA syndrome (Anomalous Left Coronary Artery Origin from the Pulmonary Artery) [49]. Between 80 and 85% of patients fail to develop adequate myocardial collateral supply and present with signs and symptoms consistent with heart failure.

Finally, collagen vascular disease needs to be ruled out in dilated cardiomyopathy patients. Dermatomyositis [50], systemic lupus erythematosus [51], rheumatic fever [52], Kawasaki disease [53], and rheumatoid arthritis [54] can all cause this condition.

The etiology of hypertrophic cardiomyopathy includes maternal diabetes; child primary hyperinsulinism or acromegaly; Pompe, Danon, or Cori–Forbes diseases; Noonan and Costello syndromes; mucopolysaccharidosis; and several fatty acid oxidation disorders [55].

### 5.4. Clinical Picture

Ventricular dilatation and systolic dysfunction are often unapparent in the preclinical phase of dilated cardiomyopathy [56], or the patient may experience palpitations, explained by arrhythmias or conduction defects [4].

The symptoms that older children experience in the initial phases are nonspecific and include fatigability, effort-related dyspnea, cough, palpitations, thoracic pain, pre-syncope, and syncope [57]. Infants and toddlers may be brought for examination because of symptoms such as irritability, poor feeding, excessive sweating during alimentation, and failure to thrive, or symptoms of heart failure-related peripheral organ dysfunction, such as oliguria. Other possible symptoms include hemoptysis, abdominal pain, and wheezing [58]. Wheezing is explained by terminal bronchiolar extrinsic compression by excess interstitial fluid in the decompensated heart failure phase.

Clinical examination reveals signs of left and right ventricular systolic dysfunction. Diminished anterograde left ventricular flow leads to cool extremities, weak peripheral pulses, and hypotension. Right ventricular failure manifests as peripheral edema, hepatomegaly, stasis crepitations, increased jugular pressure, or hepato-jugular reflux. Gallop rhythm, defined by the presence of the third or fourth heart sound, may also be present, as may murmurs of tricuspid or mitral regurgitations. These murmurs are explained not by valvular disease but by heart chamber dilatation, which eventually involves all four cavities, leading to annular dilatation. Murmurs may go unnoticed if the patient is in shock or acute heart failure because severe contractility impairment causes less blood to flow back to the atria.

Tachycardia is not only another important clinical sign but also a central compensatory mechanism. In most of these patients, the other classical central compensatory mechanism, the increase of the ventricular contraction force, is unavailable.

When ALPACA syndrome causes myocardial dysfunction, children may present with typical angina or a continuous murmur on the left parasternal border caused by blood flow through collateral vessels [49].

In hypertrophic cardiomyopathy, children may present with symptoms of congestive heart failure, angina, diminished anterograde outflow symptoms such as dizziness, presyncope or syncope, and sudden death caused by ventricular fibrillation. Orthopnea and paroxysmal nocturnal dyspnea are caused by diastolic dysfunction and pulmonary venous congestion. Whenever the clinician notices a systolic ejection murmur that has a crescendo–decrescendo character, an echocardiogram is indicated. The murmur described above may be accompanied by another one—one of mitral regurgitation, best heard at the apex or in the axilla, or one of aortic regurgitation, which is diastolic.

### 5.5. Echocardiography

Echocardiography is a reliable diagnostic tool for both dilated and hypertrophic cardiomyopathy, as well as most heart malformations.

Basic measurements in suspected cases of dilated cardiomyopathy include wall thickness and chamber dimensions (including the end-systolic and end-diastolic diameters of the left ventricle) and chamber volumes (including the end-systolic and end-diastolic volumes of the left ventricle). Systolic function is estimated through the ejection fraction and shortening fraction. The ejection fraction can be visually estimated—a method that is no longer recommended because of interobserver variability—or estimated using the Teicholz, one-plane, modified one-plane, and biplane Simpson methods. Mitral and tricuspid annular plane systolic excursions also provide information about longitudinal shortening. The pressure increase rate inside the left ventricle can be estimated on the basis of a mitral regurgitation envelope—if it exists—using the dp/dt ratio. Global ventricular function, both systolic and diastolic, can be evaluated using the Tei index. It is not dependent on age, heart rate, or left ventricle geometry, but it is dependent on afterload [59].

Regional systolic kinetics is evaluated using the Wall Motion Score Index or newer methods, such as harmonic imaging, acoustic quantification, color kinesis, contrast echography, tissue Doppler imaging, and myocardial strain/strain rate [60].

Diastolic dysfunction in dilated cardiomyopathy is also possible. Ways to estimate the severity of the dysfunction include the diastolic trans-mitral flow (E/A >2, E wave deceleration time <150 ms, isovolumetric relaxation time <60 ms), Doppler flow in the pulmonary veins (the S wave diminishes and the AR wave becomes larger), and tissue Doppler imaging [60].

In hypertrophic cardiomyopathy, the echocardiogram must focus on correct wall thickness measurement and left ventricular outflow tract velocity measurement. This last measurement, along with the systolic anterior motion of the anterior mitral valve leaflet, differentiates between the obstructive and nonobstructive forms of the disease. Furthermore, left ventricular diastolic patterns must be carefully interrogated in order to diagnose diastolic dysfunction.

### 5.6. Histological Findings

In children, it is rarely indicated to obtain a biopsy specimen via endomyocardial biopsy (unless the clinician suspects cardiac involvement in a systemic disease). Biopsy on necropsy findings of dilated cardiomyopathy patients includes myocyte atrophy [61], myocyte vacuolar degeneration [62], myositis [62], nuclear pleomorphism [63], focal fat or inflammatory infiltration, and fibrosis [64].

Aspects encountered in hypertrophic cardiomyopathy include disorganization of the myofibrillar architecture, visible fibrosis, and abnormal intramural coronary arteries, explaining chest pain in children.

### 5.7. Treatment

Children with dilated cardiomyopathy should be encouraged to remain active whenever possible, as physical activity improves muscle and mental tonus and life quality. Diet should take into consideration the hypercatabolic state associated with this condition, as well as the fact that some children may be too sick for oral feeding. In this group, nasogastric tube or intravenous feeding is necessary.

Whenever the cause of the disease is known, etiological treatment should be attempted whenever possible. Symptomatic treatment addresses the heart failure manifestation and consists of angiotensin-converting enzyme inhibitors (which have been shown to reduce mortality in adults), diuretics and beta-blockers (under close supervision, so that they do not worsen symptoms), or even ivabradine [64]. Intracardiac thrombi or spontaneous contrast dictate the need for chronic anticoagulation [65]. Digoxin, the classical oral positive inotropic agent, is currently seen as second-line therapy [65]. Intractable cardiac failure requires left ventricular or biventricular assist devices as a bridge to heart transplantation.

Hypertrophic cardiomyopathy patients should receive medication in order to reduce the left ventricle outflow gradient, such as beta-blockers. Calcium channel blockers, amiodarone, and disopyramide are effectively used in adults to treat and prevent ventricular arrhythmias [11]. Inotropic drugs, nitrates, and sympathomimetic amines should be avoided whenever possible. Some children, those who have suffered from ventricular fibrillation, ventricular tachycardia, or cardiac arrest, should be considered for implantable cardioverter defibrillators.

Heart malformations must be operated on before they cause chamber dilatation or pulmonary hypertension. In the meantime, children may receive heart failure treatment.

### 5.8. Outcomes

The prognosis of dilated cardiomyopathy seems to be worse in children than adults. The disease seems to progress faster in children, despite the fact that heart failure symptoms had been observed for a shorter period of time before diagnosis. Furthermore, children develop life-threatening ventricular arrhythmias more often than their adult peers [66] and require heart transplantation more often [12]. The prognosis is worse in those presenting with heart failure symptoms after the age of 1 year old, in those with severe dilatation and poor systolic function [67], and in those with idiopathic dilated cardiomyopathy [68]. Prognosis is better in patients with myocarditis-related dilated cardiomyopathy [69]. In some countries, heart transplantation has become more accessible over the past years. Multidisciplinary care improved patient prognosis recently [68]. One study [67,70] reported 81% of free-from-death or transplantation children at 5 years if the cause of the dilated cardiomyopathy was myocarditis, while the same parameter dropped to 62% in idiopathic or familial disease. Family history of cardiac disease and the need for inotropic support during hospitalization and other associated abnormalities were also identified as poor prognostic factors by others [71,72,73].

Heart transplantation is recommended for children with hypertrophic cardiomyopathy with restrictive physiology who do not respond to other interventions. Survival is etiology- and age-specific in these patients [74].

## 6. Conclusions

Cardiac disease needs to be thoroughly investigated using multiple tools in all children presenting with heart failure symptoms, in those with heart murmurs, and in children scheduled for surgery of any type. Cyanosis does not need to be present, since multiple potentially life-threatening heart conditions, such as dilatative or hypertrophic cardiomyopathy, are not accompanied by this symptom. A complicated medical history or the presence of abnormal findings on clinical examination should point the treating physician toward recommending a pediatric cardiologist consultation. In these instances, cardiological diagnosis is a must. Additionally, every newborn should benefit from a cardiac ultrasound and electrocardiography to rule out cardiac pathology, especially if a surgical intervention is planned for them in the future. The intensive care specialist and surgeon need to be aware of any heart pathology before non-cardiac surgical interventions; therefore, a preoperative cardiological consult may prove beneficial even if there are no obvious signs of cardiac disease.

## Figures and Tables

**Table 1:** Patient characteristics.

Sex	Age	Hospitalization Duration	Cardiomyopathy Known Before?	Cardiomegaly Known Before?	Cardiomyopathy Diagnosed during Last Hospitalization?	Cardiomegaly Diagnosed during Last Hospitalization?	Cardiomyopathy First Diagnosed during Necropsy?	Cardiomegaly First Diagnosed during Necropsy?	Underwent Surgery?	Probable Mechanism or Cause of Death
1. M	3 months	15 days	no	no	yes	no	no	no	no	Corpus callosum agenesia
2. M	1 month	7 days	no	no	yes	no	no	no	no	Spinal amyotrophy
3. M	8 months	6 days	no	no	yes	no	no	no	yes—umbilical and inguinal hernia	Postoperative
4. M	5 months	5 days	no	yes	no	no	different diagnosis	no	no	Probable arrhythmia
5. M	2 months	20 days	no	no	yes	no	no	no	no	Hemophagocytic lymphohistiocytosis
6. M	6 months	1 day	no	no	yes	no	no	no	no	Heart failure, bronchopneumonia
7. M	14 years	5 days	no	no	no	no	yes	no	no	Bronchopneumonia
8. M	4 years	28 days	no	yes	no	no	no	no	no	Brain tumor
9. M	3 years	31 days	no	no	no	no	no	yes	no	Bronchopneumonia
10. F	14 days	14 days	no	no	no	no	no	yes	yes—giant omphalocele, intestinal malrotation and occlusion	Postoperative heart and respiratory failure
11. F	1 day	1 day	no	no	no	yes	different diagnosis	no	yes—spina bifida, myelomeningocele	Postoperative

Limitations: During the 4 years included in this study, only 11 children suffering from cardiomyopathies or congestive heart failure died outside the cardiology clinic. This is a relatively small sample, making generalization difficult, and further studies are needed.

**Table 2:** Magnitude of the problem.

Year	Number of Patients Treated in the Hospital	Number of Deaths in the Hospital	Number of Patients Included in the Study
2018	25,959	100	4
2019	23,745	71	0
2020	11,154	86	5
2021	13,195	101	2

## References

[1] Wu W., He J., Shao X. (2020). Incidence and mortality trend of congenital heart disease at the global, regional, and national level, 1990–2017. Medicine.

[2] Malaisrie S.C., Pelletier M.P., Yun J.J., Sharma K., Timek T.A., Rosenthal D.N., Wright G.E., Robbins R.C., Reitz B.A. (2008). Pneumatic Paracorporeal Ventricular Assist Device in Infants and Children: Initial Stanford Experience. J. Heart Lung Transplant..

[3] Ho K.K., Pinsky J.L., Kannel W.B., Levy D. (1993). The epidemiology of heart failure: The Framingham Study. J. Am. Coll. Cardiol..

[4] Sadoh W., Osarogiagbon W. (2013). Underlying congenital heart disease in Nigerian children with pneumonia. Afr. Health Sci..

[5] Siontis K., Ommen S., Geske J. (2019). Sex, Survival, and Cardiomyopathy: Differences Between Men and Women with Hypertrophic Cardiomyopathy. J. Am. Heart Assoc..

[6] Fairweather D., Cooper L., Blauwet L. (2013). Sex and Gender Differences in Myocarditis and Dilated Cardiomyopathy. Curr. Probl. Cardiol..

[7] Donal E. (2019). EACVI recommendations on Imaging in Dilated Cardiomyopathies. Eur. Heart J. Cardiovasc. Imaging.

[8] Braunwald E. (2017). Cardiomyopathies: An overview. Circ. Res..

[9] Lee T.M., Hsu D.T., Kantor P., Towbin J.A., Ware S.M., Colan S.D., Chung W.K., Jefferies J.L., Rossano J.W., Castleberry C.D. (2017). Pediatric cardiomyopathies. Circ. Res..

[10] Colombo M.G., Botto N., Vittorini S., Paradossi U., Andreassi M.G. (2008). Clinical utility of genetic tests for inherited hypertrophic and dilated cardiomyopathies. Cardiovasc. Ultrasound..

[11] Soor G.S., Luk A., Ahn E., Abraham J.R., Woo A., Ralph-Edwards A., Butany J. (2009). Hypertrophic cardiomyopathy: Current understanding and treatment objectives. J. Clin. Pathol..

[12] Lipshultz S.E., Sleeper L.A., Towbin J.A., Lowe A.M., Orav E.J., Cox G.F., Lurie P.R., McCoy K.L., McDonald M.A., Messere J.E. (2003). The Incidence of Pediatric Cardiomyopathy in Two Regions of the United States. N. Engl. J. Med..

[13] Nugent A.W., Daubeney P.E., Chondros P., Carlin J.B., Cheung M., Wilkinson L.C., Davis A.M., Kahler S.G., Chow C.W., Wilkinson J.L. (2003). National Australian Childhood Cardiomyopathy Study. The epidemiology of childhood cardiomyopathy in Australia. N. Engl. J. Med..

[14] Arola A., Jokinen E., Ruuskanen O., Saraste M., Pesonen E., Kuusela A.L., Tikanoja T., Paavilainen T., Simell O. (1997). Epidemiology of idiopathic cardiomyopathies in children and adolescents: A nationwide study in Finland. Am. J. Epidemiol..

[15] Elkilany G., AL-Qbandi M., Sayed K., Kabbash I. (2008). Dilated Cardiomyopathy in Children and Adults: What is New?. Sci. World J..

[16] Hsu D., Lamour J., Canter C., Canter C.E., Kirklin J.K. (2008). Pediatric Heart Transplantation. ISHLT Monograph Series 2, Heart Diseases Leading to Pediatric Heart Transplantation: Cardiomyopathies and Congenital Heart Diseases.

[17] Mestroni L., Brun F., Spezzacatene A., Sinagra G., Taylor M. (2014). Genetic causes of dilated cardiomyopathy. Prog. Pediatr. Cardiol..

[18] Wilbert S. (2018). Aronow. Management of cardiac hemochromatosis. Arch. Med. Sci..

[19] von Hoff D.D., Layard M.W. (1979). Risk factors for doxorubicin-induced congestive heart failure. Ann. Intern. Med..

[20] Goldberg M.A., Antin J.H. (1986). Cyclophosphamide cardiotoxicity: An analysis of dosing as a risk factor. Blood.

[21] Mahrholdt H., Wagner A., Deluigi C., Kispert E., Hager S., Meinhardt G., Vogelsberg H., Fritz P., Dippon J., Bock C. (2006). Presentation, patterns of myocardial damage, and clinical course of viral myocarditis. Circulation.

[22] Molina K., Garcia X., Denfield S., Fan Y., Morrow W., Towbin J., Frazier E., Nelson D. (2013). Parvovirus B19 myocarditis causes significant morbidity and mortality in children. Pediatric Cardiol..

[23] Comar M., D’Agaro P., Campello C., Poli A., Breinholt J., Towbin J., Vatta M. (2009). Human herpes virus 6 in archival cardiac tissues from children with idiopathic dilated cardiomyopathy or congenital heart disease. J. Clin. Pathol..

[24] Talsma M., Kroos M., Visser G., Kimpen J., Niezen K. (2002). A rare presentation of childhood pompe disease: Cardiac involvement provoked by Epstein-barr virus infection. Pediatrics.

[25] Camargo P., Okay T., Yamamoto L., Del Negro G., Lopes A. (2011). Myocarditis in children and detection of viruses in myocardial tissue: Implications for immunosuppressive therapy. Int. J. Cardiol..

[26] Bowles N., Ni J., Kearney D., Pauschinger M., Schultheiss H., McCarthy R., Hare J., Bricker J., Bowles K., Towbin J. (2003). Detection of viruses in myocardial tissues by polymerase chain reaction. Evidence of adenovirus as a common cause of myocarditis in children and adults. J. Am. Coll. Cardiol..

[27] Matsumori A., Shimada T., Chapman N., Tracy S., Mason J. (2006). Myocarditis and heart failure associated with hepatitis c virus infection. J. Card. Fail..

[28] Bratincsak A., El-Said H., Bradley J., Shayan Grossfeld P., Cannavino C. (2010). Fulminant myocarditis associated with pandemic H1N1 influenza a virus in children. J. Am. Coll. Cardiol..

[29] Singh P., Hemal A., Agarwal S., Kumar D. (2015). Cardiac manifestations in HIV infected children. Indian J. Pediatrics.

[30] Schultheiss H., Kühl U., Cooper L. (2011). The management of myocarditis. Eur. Heart J..

[31] Blauwet L., Cooper L. (2010). Myocarditis. Prog. Cardiovasc. Dis..

[32] Canter C., Simpson K. (2014). Diagnosis and treatment of myocarditis in children in the current era. Circulation.

[33] Shibazaki S., Eguchi S., Endo T., Wakabayashi T., Araki M., Gu Y., Imai T., Asano K., Taniuchi N. (2016). Eosinophilic Myocarditis due to Toxocariasis: Not a Rare Cause. Case Rep. Cardiol..

[34] Pergola G., Cascone A., Russo M. (2010). Acute pericarditis and myocarditis by Toxoplasma gondii in an immunocompetent young man: A case report. Infez. Med..

[35] Hidron A., Vogenthaler N., Santos-Preciado J.I., Rodriguez-Morales A.J., Franco-Paredes C., Rassi A. (2010). Cardiac involvement with parasitic infections. Clin. Microbiol. Rev..

[36] Briana L., Scott Jennifer I., Sherwin Rehder K., Campbell M., Ozment C. (2018). Histoplasmosis Myocarditis in an Immunocompetent Host After a Recreational Mud Run. Pediatrics.

[37] Radu C., Camarasan A., Podila C., Perju-Dumbrava D. (2018). Sudden Death of a Teenager Caused by Actinomyces israelii: A Case Report. Iran. J. Public Health.

[38] Reuss C., Hall M., Blair J., Yeo T., Leslie K. (2004). Endocarditis Caused by Coccidioides Species. Mayo Clin. Proc..

[39] Feng J., Schaus B.J., Fallavollita J.A., Lee T.C., Canty J.M. (2001). Preload induces troponin I degradation independently of myocardial ischemia. Circulation.

[40] Kaspar R., Allen H., Montanaro F. (2009). Current understanding and management of dilated cardiomyopathy in Duchenne and Becker muscular dystrophy. J. Am. Acad. Nurse Pract..

[41] Hanson E., Sheldon M., Pacheco B., Alkubeysi M., Raizada V. (2019). Heart disease in Friedreich’s ataxia. World J. Cardiol..

[42] Boffi L., Russo P., Limongelli G. (2018). Early diagnosis and management of cardiac manifestations in mucopolysaccharidoses: A practical guide for paediatric and adult cardiologists. Ital. J. Pediatr..

[43] Linhart A., Arad M., Elliott P., Caforio A., Pantazis A., Adler Y. ESC Working Group on Mocardial and Pericardial Diseases: Key Messages on Diagnosis and Management of Cardiac Manifestations in Anderson Fabry Disease and Glycogen Storage Diseases. https://www.escardio.org/static-file/Escardio/Subspecialty/Working%20Groups/Myocardial%20and%20Pericardial%20Diseases/y.%20Documents/Booklet-WG-Diseases-Fabry&Glycogen.pdf.

[44] Cryer M., Napier R., Compton C. (2019). Pheocromocitoma induced cardiomyopathy. J. Am. Coll. Cardiol..

[45] Rastogi P., Dua A., Attri S., Sharma H. (2018). Hypothyroidism-induced reversible dilated cardiomyopathy. J. Postgrad. Med..

[46] Al-Ghamdi A., Aljohani N. (2013). Graves’ Thyrotoxicosis-Induced Reversible Cardiomyopathy: A Case Report. Clin. Med. Insights Case Rep..

[47] Babu M., Sameer S. (2011). Hypoparathyroidism and reversible dilated cardiomyopathy. Indian J. Endocrinol. Metab..

[48] Byers S., Ficicioglu C. (2014). Infant with cardiomyopathy: When to suspect inborn errors of metabolism?. World J. Cardiol..

[49] Rodriguez-Gonzalez M., Tirado A., Hosseinpour R., de Soto J.S. (2015). Anomalous Origin of the Left Coronary Artery from the Pulmonary Artery: Diagnoses and Surgical Results in 12 Pediatric Patients. Tex. Heart Inst. J..

[50] Schwartz T., Pyndt Diederichsen L., Lundberg I., Sjaastad I., Sanner H. (2016). Cardiac involvement in adult and juvenile idiopathic inflammatory myopathies. RMD Open.

[51] Usalan C., Buyukhatipoglu H., Tiryaki O. (2007). Systemic lupus erythematosus complicated by dilated cardiomyopathy and severe heart failure. Clin. Rheumatol..

[52] Amritanshu K., Banerjee D. (2013). Concomitant Rheumatic Fever and Dilated Cardiomyopathy: A Case Report. Pediatric Oncall.

[53] Gordon J., Kahn A., Burns J. (2009). When children with Kawasaki disease grow up: Myocardial and vascular complications in adulthood. J. Am. Coll. Cardiol..

[54] Giles J., Fernandes V., Lima J., Bathon J. (2005). Myocardial dysfunction in rheumatoid arthritis: Epidemiology and pathogenesis. Arthritis Res. Ther..

[55] Monda E., Rubino M., Lioncino M., Di Fraia F., Pacileo R., Verrillo F., Cirillo A., Caiazza M., Fusco A., Esposito A. (2021). Hypertrophic Cardiomyopathy in Children: Pathophysiology, Diagnosis, and Treatment of Non-sarcomeric Causes. Front. Pediatr..

[56] Law M., Cohen H., Martin A., Angulski A., Metzger J. (2020). Dysregulation of Calcium Handling in Duchenne Muscular Dystrophy-Associated Dilated Cardiomyopathy: Mechanisms and Experimental Therapeutic Strategies. J. Clin. Med..

[57] Jefferies J., Towbin J. (2010). Dilated cardiomyopathy. Lancet.

[58] Andrews R., Fenton M., Ridout D., Burch M. (2008). New-onset heart failure due to heart muscle disease in childhood: A prospective study in the United Kingdom and Ireland. Circulation.

[59] Lakoumentas J., Panou F., Kotsergolou V. (2005). The Tei index of myocardial performance. Aplications in cardiology. Helenic. J. Cardiol..

[60] Carmen Ginghina Popescu B.A., Jurcut R. (2005). Esentialul in Ecocardiografie. Med. Anataeus.

[61] Puggia I., Merlo M., Barbati G., Rowland T., Stolfo D., Gigli M., Ramani F., Di Lenarda A., Mestroni L., Sinagra G. (2016). Natural History of Dilated Cardiomyopathy in Children. J. Am. Heart Asoc..

[62] Basso C., Ronco F., Marcus F., Abudureheman A., Rizzo S., Frigo A.C., Bauce B., Maddalena F., Nava A., Corrado D. (2008). Quantitative assessment of endomyocardial biopsy in arrhythmogenic right ventricular cardiomyopathy/dysplasia: An in vitro validation of diagnostic criteria. Eur. Heart J..

[63] Johnson R., Palacios I. (1982). Dilated cardiomyopathies of the adult (second of two parts). N. Engl. J. Med..

[64] Bonnet D., Berger F., Jokinen E., Kantor P.F., Daubeney P.E.F. (2017). Ivabradine in children with dilated cardiomyopathy and symptomatic chronic heart failure. J. Am. Coll. Cardiol..

[65] Franklin O.M., Burch M. (2000). Dilated cardiomyopathy in childhood. Images Paediatr. Cardiol..

[66] Mitrut R., Stepan A., Pirici D. (2018). Histopathological Aspects of the Myocardium in Dilated Cardiomyopathy. Curr. Health Sci. J..

[67] Towbin J., Lowe A., Colan S., Sleeper L., Orav E., Clunie S., Messere J., Cox G.F., Lurie P.R., Hsu D. (2006). Incidence, causes, and outcomes of dilated cardiomyopathy in children. JAMA.

[68] Lipshultz S., Law Y., Asante-Korang A., Austin E., Dipchand A., Everitt M., Hsu D. (2019). Cardiomyopathy in Children: Classification and Diagnosis: A Scientific Statement from the American Heart Association. Circulation.

[69] Alvarez J., Orav E., Wilkinson J., Fleming L., Lee D., Sleeper L., Rusconi P., Colan S., Hsu D., Canter C. (2011). Pediatric Cardiomyopathy Registry Investigators. Competing risks for death and cardiac transplantation in children with dilated cardiomyopathy: Results from the Pediatric Cardiomyopathy Registry. Circulation.

[70] Weintraub R., Alexander P. (2017). Outcomes in Pediatric Dilated Cardiomyopathy. J. Am. Coll. Cardiol..

[71] Ciuca C., Ragni L., Hasan T., Balducci A., Angeli E., Prandstraller D., Egidy-Assenza G., Donti A., Bonvicini M., Gargiulo G. (2019). Dilated cardiomyopathy in a pediatric population: Etiology and outcome predictors—A single-center experience. Future Cardiol..

[72] Tarca E., Plamadeala P., Savu B. (2014). Plurimalformative syndrome associating trisomy 18 and omhalocele. Case report and review of the literature. RJME.

[73] Tarca E., Cojocaru E., Rosu S., Butnariu L., Plamadeala P., Moisa S.M. (2019). Differential diagnosis difficulties related to infantile hemangioma- case report and literature review. RJME.

[74] Steven C. (2010). Hypertrophic cardiomyopathy in childhood. Heart Fail. Clin..

